# No evidence for sibling or parent–offspring coadaptation in a wild population of blue tits, despite high power

**DOI:** 10.1111/evo.13642

**Published:** 2018-11-27

**Authors:** Caroline E. Thomson, Jarrod D. Hadfield

**Affiliations:** ^1^ Department of Zoology, Edward Grey Institute University of Oxford Oxford OX1 3PS United Kingdom; ^2^ Institute of Evolutionary Biology, School of Biological Sciences University of Edinburgh Edinburgh EH9 3JT United Kingdom

## Abstract

Parent and offspring behaviors are expected to act as both the agents and targets of selection. This may generate parent–offspring coadaptation in which parent and offspring behaviors become genetically correlated in a way that increases inclusive fitness. Cross‐fostering has been used to study parent–offspring coadaptation, with the prediction that offspring raised by non‐relatives, or parents raising non‐relatives, should suffer fitness costs. Using long‐term data from more than 400 partially crossed broods of blue tits (*Cyanistes caeruleus*), we show that there is no difference in mass or survival between crossed and non‐crossed chicks. However, previous studies for which the evidence for parent–offspring coadaptation is strongest compare chicks from fully crossed broods with those from non‐crossed broods. When parent–offspring coadaptation acts at the level of the brood then partial cross‐fostering experiments are not expected to show evidence of coadaptation. To test this, we performed an additional experiment (163 broods) in which clutches were either fully crossed, non‐crossed, or partially crossed. In agreement with the long‐term data, there was no evidence for parent–offspring coadaptation on offspring fitness despite high power. In addition there was no evidence of effects on parental fitness, nor evidence of sibling coadaptation, although the power of these tests was more modest.

Correlational selection occurs when the fitness function of a trait depends on the value of another trait an individual expresses. This can result in favorable genetic correlations, either through the build up of linkage disequilibria between alleles affecting the two traits (Fisher 1930; Lewontin and Kojima 1960; Lewontin 1964), or the segregation of alleles that have pleiotropic effects (Lande 1980; Cheverud 1984). When the fitness of an individual is affected by its relatives (either directly or indirectly), selection can favor the formation of genetic correlations between traits that have effects on *inclusive* fitness (Wade 1998). In the context of parents and their offspring, these genetic correlations generate parent–offspring coadaptation, in which the combination of trait values within a family result in the highest fitness (Wolf and Brodie 1998; Kölliker et al. 2005).

For parent–offspring coadaptation to exist, three things must be satisfied: (a) a set of genes in the actor must affect the fitness of the recipient, (b) this effect must depend on a set of genes in the recipient, and (c) the effects of these genes in the actor and the recipient must be correlated. Traditionally, parents are considered the actors and offspring the recipients, such that parent–offspring coadaptation is manifest as effects on offspring fitness. A necessary condition for this to happen is that heritable traits that exert parental effects exist, and are genetically correlated with the offspring traits they affect (Wolf and Brodie 1998). Although more rarely considered, offspring can also play the role of actor and coadaptation effects on parental fitness are possible when heritable traits that exert offspring effects on parents exist, and are genetically correlated with the parental traits they affect (see Hinde et al. 2010, in the context of parent–offspring signaling). Because of this, much of the work on parent–offspring coadaptation has focused on the specific traits expected to mediate parent–offspring interactions and their genetic basis. Whilst, the evidence for heritable parental effects is widespread (Räsänen and Kruuk 2007), heritable offspring effects are harder to detect, and thus the evidence is more limited (Agrawal et al. 2001). With regard to the traits mediating such effects, parental provisioning and offspring begging have been well studied, with the expectation that if provisioning affects the rate of begging (parental effect), and/or begging affects the rate of provisioning (offspring effect), genetic correlations may generate coadaptation (Kölliker et al. 2005). Positive parent–offspring correlations between provisioning and begging, attributed to genetic or prenatal effects, have been found using cross‐fostering in great tits (*Parus major*; Kölliker et al. 2000), burying beetles (*Nicrophorus pustulatus*; Lock et al. 2004), and canaries (*Serinus canaria domestica*; Hinde et al. 2009; Estramil et al. 2013), but not blue tits (*Cyanistes caeruleus*; Lucass et al. 2015a). In addition, Hinde et al. (2010) demonstrated that, in canaries, the loss of growth when offspring were cross‐fostered was related to the difference in begging intensities of the biological offspring of the foster parents, and the foster offspring themselves, suggesting that these traits may underpin any coadaptation. Lucass et al. (2015b) demonstrated that, in blue tits, the highest masses were achieved by high‐begging chicks raised by high‐provisioning foster parents, but these chicks showed the lowest masses when with low‐provisioning parents. Nevertheless, without a genetic correlation between these behaviors in blue tits (Lucass et al. 2015a) this does not constitute coadaptation.

Whilst the traits that underpin such coadaptation are of interest, coadaptation is ultimately measured through its consequences on fitness, with the expectation that individuals have higher fitness when interacting with relatives rather than non‐relatives. Hinde et al. (2010) compared the fitness of family members in fully cross‐fostered and non‐cross‐fostered nests of canaries, and were the first to interpret this comparison in the context of parent–offspring coadaptation. They found evidence for parent–offspring coadaptation through effects on offspring fitness, but not on parental fitness, although this could not be replicated (Estramil et al. 2013, 2014). Although not placed in the context of parent–offspring coadaptation, identical comparisons have been used in Columbian ground squirrels (*Spermophilus columbianus*; Murie et al. 1998) and domestic pigs (*Sus scrofa domesticus*; Heim et al. 2012) with no evidence that cross‐fostering reduces the growth or survival of offspring.

Partial cross‐fostering studies in which a subset of offspring are moved between nests and litters (Rutledge et al. 1972) allow comparisons of cross‐fostered and non‐cross‐fostered offspring “within” nests and litters. This design has been used for the purpose of testing cross‐fostering effects in Barn swallows (*Hirundo rustica*; Boncoraglio and Saino 2008) and domestic pigs (Heim et al. 2012), again with no effects on offspring growth or survival. However, this experimental design is more commonly used by quantitative geneticists to estimate genetic and postnatal maternal effects, and such studies occasionally report the difference between cross‐fostered and non‐cross‐fostered offspring. Of those that do report such effects, no differences were found for body mass in starlings (*Sturnus vulgaris*; Smith and Wettermark 1995) and burying beetles (Rauter and Moore 2002), or for survival in cliff swallows (*Petrochelidon pyrrhonota*; Brown et al. 2015). In contrast, Winney et al. (2015) showed that in house sparrows (*Passer domesticus*), cross‐fostered offspring had higher survival, but attributed this to methodological problems. The partial cross‐fostering design does not allow the consequences of parent–offspring coadaptation on parental fitness to be measured, as all parents rear a mix of related and unrelated offspring. Finally, three studies have compared offspring from non‐cross‐fostered nests/litters with non‐crossed offspring from partially crossed nests/litters. No differences in survival were found in house sparrows (Winney et al. 2015) or domestic pigs (Heim et al. 2012), but the survival of Barn swallows was found to be lower for non‐crossed chicks in mixed maternity nests (Boncoraglio and Saino 2008).

Thus, the results from these studies are mixed with the tentative observation that between‐brood comparisons show more evidence of parent–offspring coadaptation than within‐brood comparisons. This possible difference may be due to partial and fully cross‐fostered designs differing as to whether non‐siblings are raised together, which could result in differences if parental responses to offspring behavior affect all offspring in the brood equally, rather than affecting each offspring in proportion to its individual effects. For example, parents may respond to the begging behavior of individuals by increasing the total rate of food provisioning to the brood (Ottosson et al. 1997) and so an individual that begs at a high rate may gain more food for both itself and its siblings. We will call this form of parent–offspring coadaptation sibling‐mediated parent–offspring coadaptation (SPO). Under SPO, no differences between the fitness of cross‐fostered and non‐cross‐fostered offspring within partially cross‐fostering nests are expected, because parents do not vary in the average relatedness to the brood that they raise (see Figure 1). Fitness costs would still be expected, however, between fully cross‐fostered broods and controls. Additionally, siblings may also have *direct* effects on one another. These may arise when sibling behaviors, such as negotiation rules (Roulin 2002; Romano et al. 2012), increase fitness when all nest mates adopt a similar set of behaviors (Mock and Parker 1997). Should these have a genetic basis, siblings will have more similar behaviors than non‐siblings, causing chicks from partially crossed nests to have lower fitness than chicks from fully cross‐fostered or control nests, regardless of whether they have been crossed or not (see Figure 1).

**Figure 1:**
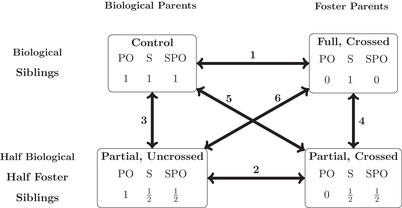
The comparisons that can be made between individuals in different types of nest generated by using full cross‐fostering, partial cross‐fostering, and randomised controls. The codes within boxes stand for parent–offspring coadaptation (PO), sibling‐mediated parent–offspring coadaptation (SPO), and sibling coadpatation (S). The numbers underneath each code represent the extent to which individuals gain the fitness benefits of being with relatives through each process, compared with controls. Under certain genetic models, these numbers will be proportional to the relatedness of the individual to the adult that raises it (PO) the relatedness of the individual's nest‐mates to the adult that raises it (SPO) and the relatedness of the individual to its nest mates (S).

Here, we present the results of a randomized experiment that compares offspring and parental fitnesses across fully cross‐fostered, partial cross‐fostered, and non cross‐fostered broods of blue tits. The design allows us to estimate the magnitude of parent–offspring (PO) coadaptation, SPO coadaptation, and sibling (S) coadaptation. Under parent–offspring coadaptation (PO), we expect that cross‐fostered offspring should have lower fitness than those that are not crossed, as they are raised by non‐related parents. Under SPO coadaptation, although we expect that there will be a fitness cost to crossed offspring, this will be of smaller magnitude in partially cross‐fostered nests than in fully cross‐fostered nests. In addition a cost should also be felt by non‐crossed offspring in partially crossed nests. If sibling coadaptation is present, there should be no fitness costs to being cross‐fostered in the full treatment, but costs to both crossed and non‐crossed offspring in the partial treatment (as they are raised with non‐siblings).

## Methods

This study was carried out in a nest‐box population of blue tits on the Dalmeny estate in Edinburgh, UK. Blue tits are small sedentary passerines that usually start to breed in their first summer and lay a single large clutch each year (a mean of 8.9 eggs in our population). Both parents provision the chicks with invertebrates, preferentially lepidoptera larvae if possible, but consume a large range of animal and plant matter when adults. Adult mortality is high for a bird, with the annual survival probability being approximately a half (47% in our population). See Perrins (1979) for an excellent summary of their life history.

This population has been studied since 2009, and consists of 180 boxes on Craigie Hill (grid reference NT156766) and 46 beside the Almond River (NT179758), spaced around 30 m apart (Hadfield et al. 2013a, b; Thomson et al. 2017). The experiment was conducted in the breeding seasons of 2014 and 2015, although we also utilize data collected between 2010 and 2013, and 2016 and 2017. Nest boxes were visited systematically from early April, such that occupied boxes were known in advance of egg laying, and the first egg was found on the day that it was laid.

### CROSS‐FOSTERING

**2014 and 2015**: Nests were paired randomly with other nests in which the first egg was laid on the same day, and assigned to cross‐fostering treatments. Pairs of nests were drawn at random and assigned sequentially to treatments resulting in a balanced design with a uniform distribution of treatments across the breeding season. There were three treatments: control, partial cross‐fostering, and full cross‐fostering. Control nests did not have any eggs exchanged between them, such that all chicks were raised in their nest‐of‐origin (30 nests in 2014, 32 in 2015). For partial cross‐fostered nests, we aimed to reciprocally exchange every other egg between the pair of nests, so the first egg was exchanged, the second was not, the third was, and so on, such that approximately half of the eggs would be crossed (26 nests in 2014, 34 in 2015). For fully cross‐fostered nests, we aimed to reciprocally exchange every egg between the nests (28 nests in 2014, 34 in 2015). Eggs were exchanged on the day they were laid in both treatments. On some days, an odd number of nests commenced laying, such that one nest on that day could not be assigned to a pair. If the nest would have been assigned a control treatment (had a second nest to pair with been available), this treatment was assigned and the second nest in the pair assigned the following day. However, pairs could not be generated for nests starting on different days in the full or partial treatment, so the unmatched nest was assigned as an “odd” nest, in which no eggs were crossed but it was not in a matched pair (21 nests in 2014, 14 in 2015). The position in the laying sequence was written on each egg using a nontoxic marker.

**Figure 2:**
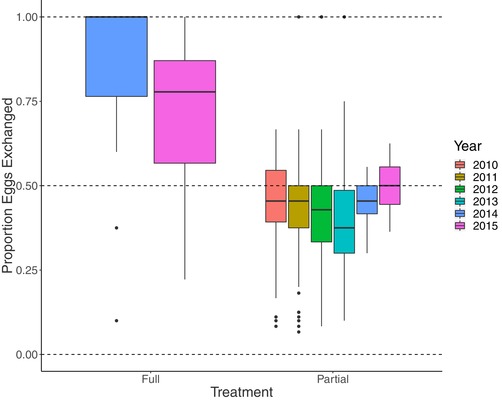
The proportion of eggs crossed in each nest within the treatment categories. No eggs were ever exchanged between nests classed as Control or Odd. Boxes are shaded by year. Only 2014 and 2015 included the full treatment as part of the experimental design.

Blue tits often pause during laying for one or more days, such that there is an interruption in the laying sequence. We did not wish to alter clutch (and subsequently brood) sizes, as these are known to have effects on both chicks and parents (Sanz 1997; Neuenschwander et al. 2003; De Heij et al. 2006; Parejo and Danchin 2006), so egg exchanges were not carried out if one of the pair did not lay an egg on a given day. Similarly, crossing of eggs ceased when one of the nests in the pair ceased laying and started incubating eggs. Thus the partial and full cross‐fostering treatments have some variation in the proportion of eggs actually crossed between nests, shown in Figure 2.

**2010 to 2013**: During these years all nests were assigned to cross‐fostering groups on the day that the first egg was laid, with a partial cross‐fostering design being used for all nests. Where possible, nests were assigned into cross‐fostering groups of three, and eggs were exchanged in a round‐robin design: the egg from nest A was transferred to nest B, that from nest B to nest C, and that from nest C to nest A. Although most nests were in triads, some groups contained two, four, or five nests, if the number of nests that commenced laying on a given day was not divisible by three. As with the partial treatment above, eggs were crossed every other day, so that approximately half of all eggs were crossed between nests (Figure 2). In total, there were 489 partial nests. There were also 35 nests that could not be assigned to cross‐fostering groups and these were classed as “odd” rather than controls, as they also tended to lie at the extreme ends of the laying period. These data were included as they increase the power of the model and improve the estimation of the variance components included. In 2010–2011, an additional cross‐fostering experiment was carried out in which first or last laid eggs within nests were exchanged between nests that were not already in the same cross‐fostering group. In 2012–2013, an additional experiment was carried out in which some parents were provided with the larvae of the wax moth (*Gralleria mellonellafed*) during different stages of the laying period. Treatments in 2010–2011 had no significant effect on postnatal development (Hadfield et al. 2013b) and treatments in 2012‐2013 had no significant effect on prenatal development (Thomson and Hadfield 2017), so we ignore these treatments here (see Thomson et al. 2017 also).

**2016‐2017**: During these years, nests were subject to the round‐robin cross fostering design described above, and in 2017, a begging playback experiment was conducted. However, for this study, data from these years were only used to obtain the following year's fecundity of parents breeding in 2015 (2016) and to assess their survival (primarily 2016, although four parents from 2015 were first recaught in 2017).

### DATA COLLECTION

Females were classed as having completed laying and having commenced incubation once eggs were no longer being laid, and she was found incubating, or eggs were warm for the second day in a row. Nests were checked for hatching daily from around 11 days after the final egg had been laid. In the majority of cases this meant that chicks were found on the first day that any hatched. Due to nest failures between the start of laying and chicks hatching, the numbers of nests in each treatment that have chicks were lower than initially assigned and these numbers are summarized in Table 1 together with the numbers of chicks in each treatment.

**Table 1:** The number of chicks and nests‐of‐rearing in each treatment category split into experimental years (2014‐2015) and non‐experimental years (pre‐2013)

	Experimental years		Non‐experimental years	
Treatment	Chicks	Nests	Chicks	Nests
Control	416	53		
Partial uncrossed	243	56	2035	437
Partial crossed	206		1425	
Full crossed	315	54		
Full uncrossed	86			
Odd	211	28	157	23

The first day on which any chick hatched (Day 0) they were uniquely marked by clipping tufts of down on their head, and a toenail if necessary. These chicks were weighed (to within 0.01 g), and the numbers of any unhatched eggs were noted. The same was done on two subsequent visits (day 1 and day 3, no eggs hatched past this point). Chicks were then weighed again on four more days (days 6, 9, 12, and 15), and on each visit all mortality was noted. In addition, blood samples were taken from chicks under home office license from the medial metatarsal vein on day 3, and chicks were ringed on day 9. All nests were visited on day 25 or after to obtain fledging success.

From day 10 onward, adults were caught in the nest box, and if necessary by mist net. If the adult had not been previously caught, it was ringed, with both a metal and a color ring, which indicated the year they were first caught as adults, as well as their sex. All adults were weighed, and we took blood samples from the ulna vein.

### GENOTYPING AND PEDIGREE

Genotypes were required in order to determine the nest‐of‐origin of each chick, as the exact egg that an individual hatched from was not known in most cases. These genotypes also allowed reconstruction of the pedigree. DNA was extracted from blood samples taken from chicks and adults using DNeasy Blood, and from tissue samples of some chicks or unhatched eggs using Tissue kit (Qiagen, Hilden, Germany), and genotyped at seven polymorphic microsatellite markers (Olano‐Marin et al. 2010). See Hadfield et al. (2013a) for full molecular methods. The sex of each chick was also determined, by amplifying the sex‐linked markers P2 and P8 (Griffiths et al. 1998).

Initially, chicks were assigned to a nest‐of‐origin by simultaneously estimating the maternity and the true genotypes using MasterBayes (Hadfield et al. 2006). Nest boxes in which a female was not caught were assigned dummy mothers with missing genotypes, essentially allowing the algorithm to group siblings with unsampled mothers. Natural mixed maternity clutches were assumed not to occur, and so maternity for each chick was restricted to the females associated with the nests in its cross‐fostering group. In all cases the nest‐of‐origin could be assigned. Once the nest‐of‐origin was assigned both maternity and paternity were estimated using an approximation for dealing with missing or erroneous genotypes. Nest boxes in which a male was not caught were assigned dummy males. The analysis also included (a) the probability of extra pair mating and how it declines with distance from the nest‐of‐origin, (b) the probability that a bird known to be alive in that year but only caught in subsequent years gains parentage, and how it declines with the distance between the nest‐of‐origin and the nest at which it was caught, and (c) the probability that a dummy male gained parentage over a sampled male. Chicks that had greater than 50% posterior probability of being sired by an unsampled male were then grouped into paternal sibships using Colony (Wang and Santure 2009; Hadfield et al. 2013a).

### STATISTICAL METHODS

#### Chick mass

The weights of chicks across ontogeny were analyzed using mixed‐effects models implemented in ASReml‐R (Gilmour et al. 2009) in R (R Development Core Team 2012). Nest age was fitted as a seven‐level factor representing the day since hatching the nest was visited. In addition, we included year, sex, and hatch day (day of hatching within the nest relative to the first chick; 0, 1, or 3) as factors, and clutch size, hatch date (days since April 1st from when the first chick in the nest hatched), and time of measurement (since midnight in units of days) as continuous fixed effects. All fixed terms, including treatment (see below), were also fitted as interactions with day (nest age as a continuous variable) to capture any changes in their effects over ontogeny. Wald tests were used to jointly test each main effect and interaction pair in order to test whether there are effects of a predictor at any point during development.

A set of biologically motivated contrasts were set up to capture differences between the four treatment groups (control, full, partial crossed, and partial uncrossed). This resulted in three predictors: parent–offspring (PO) coadaptation, SPO coadaptation, and sibling coadaptation, the values of which are shown for each treatment group in Figure 1. The coefficients associated with these predictors measure the amount of coadaptation that exists by each process when comparing a control nest to the situation in which all individuals (foster parents and offspring) are mutually unrelated. Thus, positive coefficients from the model indicate family coadaptation, and negative coefficients indicate family maladaptation.

Figure 1 also shows the different comparisons that can be made between chicks exposed to different treatments. Generally, only comparisons 1 and 2 from this figure are commonly considered, which are unable to distinguish between the three types of effect: in comparison 1 (e.g., Hinde et al. 2010) PO and SPO coadaptation are confounded, and in comparison 2 (e.g., Smith and Wettermark 1995) only PO coadaptation can be estimated. Comparisons 3 and 5 have also been made (Boncoraglio and Saino 2008) but here sibling coadaptation and SPO are confounded. Our design allows additional comparisons that can distinguish all three types of coadaptation (see Heim et al. 2012, also): If PO coadaptation exists, both crossed chicks in full and partial treatments should suffer because they are not related to their foster parents (comparisons 1, 2, and 5). If SPO coadaptation is important, however, crossed and uncrossed individuals in the partial treatment should be identical, but suffer less than crossed individuals in full treatments and suffer more than uncrossed controls (comparisons 3 and 5 vs. 4 and 6). This occurs because the “mean” relatedness of parents to the chicks they raise is approximately 0.25, 0, and 0.5 in partial, full, and control nests, respectively. Finally, if sibling coadaptation is important then individuals in partial nests should do worse than those in control and full nests (comparisons 3 and 4) because they are only related to half their nest‐mates, rather than all.

Winney et al. (2015) showed that cross‐fostering studies can produce results contrary to expectations due to nonrandom assignment of chicks to treatments. This is likely to be the case for nests not assigned to any treatment (odd nests), as they could not be placed in appropriate cross‐fostering groups, and these tended to occur at extreme ends of the laying season compared to controls. Individuals in these nests got the same predictor as control nests, but an additional fixed effect (odd nests = 1, all other nests = 0) was added to account for any bias. In the full treatment, any uncrossed eggs were given 1 for PO coadaptation (as they remained with their biological parents) and 0 for sibling and SPO coadaptations, under the assumption that clutch sizes were large and there were few such chicks. However, uncrossed eggs are those laid when the other member of the pair did not lay (eggs laid late in the sequence in the larger of the two clutches and/or in nests in which the paired nest had a laying interruption) and so again we fitted a unique fixed effect for uncrossed chicks in the full treatment to control for any bias. In addition, the differences in experimental design between 2010–2013 and 2014–2015 might cause systematic differences between odd nests, between the time periods and partial nests, and between the time periods (there were no controls or full treatment in 2010–2013). In order to include the information gained by comparing crossed and uncrossed chicks in partial nests from 2010–2013, we fitted terms that allowed odd and partial nests to differ between the two time periods. Those bias‐correction terms (and their interactions with nest age) that were nonsignificant were dropped from the main model for clarity.

In the partial treatment, eggs were allocated to be crossed or not‐crossed alternately through the laying sequence. However, if a female pauses then this allocation breaks down for subsequent eggs, which could lead to biases. Unfortunately, it was not possible to statistically exclude chicks that come from these subsequent eggs, as was done with uncrossed chicks from the full‐cross‐fostering treatment. This was because chicks could not be uniquely assigned to eggs and so it was not possible to distinguish chicks from the same half‐brood that come from eggs laid before or after the point at which the switching design was disrupted. As with the full treatment, late laid eggs in large clutches were more likely to be uncrossed and these eggs were likely to hatch later, and thus have reduced mass and survival (Hadfield et al. 2013b). Fitting hatch day as a fixed effect in this model was used to try to eliminate this effect. However, a correlation between egg rank and hatching times (and therefore treatment and hatching times) may remain within a given hatch day. To try and correct for this, we defined egg rank as the time in days between a given egg being laid and the last egg in the nest‐of‐rearing being laid, such that the last egg had rank zero, the penultimate egg rank one, and so on. We then took the average rank of eggs a chick could have hatched from (based on timing and nest‐of‐origin) and fitted this as a covariate. The mean rank of crossed and uncrossed eggs in partial nests was 5.373 and 3.925, respectively, and 4.819 and 2.638, respectively, in full crossed nests. As with other fixed effects, this was included in the model, along with an interaction with nest age.

We included three random effects in the model, all fitted as a 7× 7 variance covariance matrix across the seven nest ages. Genetic (pedigree) and nest‐of‐origin random effects were approximated using a first order autoregressive structure (Hadfield et al. 2013a), whereas nest‐of‐rearing was fitted as an unstructured matrix. Residual mass effects were also unstructured.

#### Chick survival

The survival of chicks were analyzed in a mixed‐effects model in MCMCglmm (Hadfield 2010) in R (R Development Core Team 2012). Survival of each chick was fitted as a repeat‐measure binary trait, as in event‐history analysis, using a probit link function. The fixed effects were the same as for the mass models, including the interaction of effects with day. Nest‐of‐rearing was fitted as a random effect, as was the interaction between nest‐of‐rearing and nest age, to account for the fact that all chicks within a nest often suffer mortality at the same age due to parental desertion. Flat priors were used for all random effects (zero degree of belief in the inverse‐Wishart prior), except for the residual variance, which was fixed at 1. Individual effects to account for overdispersion as in Hadfield et al. (2013a), were not fitted because they are confounded with the fixed age effects and therefore information about their variance depends solely on the prior.

#### Adult survival and fecundity

The effects of the treatments on adults were fitted in MCMCglmm. Treatment was fitted as a fixed effect in these models, as the predictions of the effect of cross‐fostering under parent–offspring coadaptation and sibling‐mediated parent–offspring coadaptation were identical, and so these could not be fitted as separate predictors. Although the model cannot take into account variation in recapture probability, adult recapture probabilities are high in this population, such that the probabilities of correctly scoring a surviving bird were 0.95 (females) and 0.94 (males) if there was no permanent emigration of adults (there is low movement of adults between areas within the field site).

Log fecundity was fitted as a Gaussian trait, and the effect of treatment was fitted separately for each sex within the model. Residual variances were also fitted separately for the sexes, with flat priors as above. Survival was fitted as a binary variable with probit link, again with the effects fitted separately for each sex, and the residual variance fixed at 1 in the prior.

## Results

### CHICK MASS

**Figure 3:**
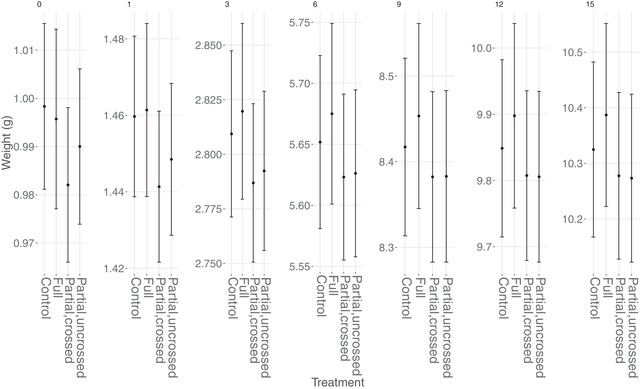
Predicted means and standard errors for chick weights in each of the main treatment categories. All continous covariates were set to their average values, and the categorical predictors were Female for Sex, 2010 for year, and (0–1) for hatch day. Note that the standard errors do not reflect the uncertainty around the treatment *differences*, particularly for crossed and uncrossed chicks in partially treated nests, in which the sampling errors are strongly correlated (as crossed and uncrossed chicks are within the same nest, and resemble each other due to nest‐of‐rearing effects).

The fixed effects of the model of chick mass are summarized in Table 2. There was an overall nonsignificant effect of PO coadaptation (Wald test *P* = 0.065) in the opposite direction to what might be expected—there was a nonsignificant reduction in mass at day 0 when with biological parents rather than foster parents (–0.005 ± 0.004 g, *P* = 0.158), equivalent to a 0.53% loss of mass; this difference was suggestive at day 15 (–0.058 ± 0.026 g, *P* = 0.027), but again this is only equivalent to a 0.54% reduction in mean mass at this age. There was no significant effect of SPO coadaptation (Wald test *P* = 0.931; 0.008 ± 0.022 g, *P* = 0.719 at day 0; –0.004 ± 0.202 g, *P* = 0.983 at day 15). Likewise, there was no overall effect of sibling coadaptation (Wald test *P* = 0.237; 0.022 ± 0.016 g, *P* = 0.176 at day 0; 0.161 ± 0.117 g, *P* = 0.171 at Day 15). In order to visualize the PO, SPO, and sibling effects in terms of treatments, the predicted mean nest weights for each treatment at each nest age are shown in Figure 3, with chicks from partial nests split into those who have and have not been cross‐fostered.

**Table 2:** Fixed effect results for the model of weight across ages in all years. The mechanisms by which crossed offspring may lose fitness have been fitted as parent–offspring coadaptation (PO), sibling coadaptation (S), and sibling‐mediated parent–offspring coadaptation (SPO). In addition, odd nests were fitted as a separate factor, as were uncrossed individuals in the full treatment

	Estimate	SE	Pr(>|Z|)	Pr(>W)
Day 0	0.560	0.058	<0.001	
Day 1	1.116	0.069	<0.001	
Day 3	2.655	0.121	<0.001	
Day 6	5.782	0.220	<0.001	
Day 9	8.832	0.323	<0.001	
Day 12	10.548	0.425	<0.001	
Day 15	11.309	0.526	<0.001	
Time	0.559	0.032	<0.001	
Time: Day	0.131	0.012	<0.001	<0.001
Sex (M)	0.006	0.003	0.034	
Sex (M): Day	0.033	0.001	<0.001	<0.001
Year 2011	−0.027	0.015	0.080	
Year 2011: Day	−0.034	0.009	<0.001	<0.001
Year 2012	−0.026	0.015	0.089	
Year 2012: Day	−0.017	0.009	0.055	0.053
Year 2013	−0.090	0.019	<0.001	
Year 2013: Day	−0.013	0.012	0.256	<0.001
Year 2014	0.032	0.017	0.063	
Year 2014: Day	−0.014	0.010	0.150	0.044
Year 2015	−0.080	0.017	<0.001	
Year 2015: Day	−0.029	0.010	0.003	<0.001
Clutch Size	−0.005	0.003	0.041	
Clutch Size: Day	−0.002	0.002	0.175	0.057
Hatch Date	0.004	0.001	<0.001	
Hatch Date: Day	−0.003	0.000	<0.001	<0.001
Hatch Day (0‐1)	−0.168	0.006	<0.001	
Hatch Day (0‐1): Day	−0.026	0.003	<0.001	<0.001
Hatch Day (1‐3)	−0.716	0.026	<0.001	
Hatch Day (1‐3): Day	−0.038	0.006	<0.001	<0.001
Egg Rank	0.003	0.001	0.011	
Egg Rank: Day	−0.000	0.001	0.698	0.033
Odd nest	−0.083	0.021	<0.001	
Odd nest: Day	−0.014	0.013	0.272	<0.001
PO	−0.005	0.004	0.158	
PO: Day	−0.003	0.002	0.037	0.065
S	0.022	0.016	0.176	
S: Day	0.009	0.008	0.222	0.237
SPO	0.008	0.022	0.719	
SPO: Day	−0.001	0.013	0.951	0.931

Odd nests were significantly lighter than nests assigned to treatments (Wald test *P* < 0.001), although this was present early in ontogeny but not later (–0.083 ± 0.021 g, *P*< 0.001 at day 0; –0.290  ±  0.193 g, *P* = 0.132 at day 15). No differences were found between crossed and non‐crossed chicks in the full treatment, nor were differences found between between experimental (2014–2015) and non‐experimental years (2010–2013) and so these bias‐correction terms were dropped (results not shown).

In addition, there were significant effects of time, sex, year, hatch date, and hatch day within the nest (Table 2). Egg rank had a suggestive positive effect on mass early in ontogeny (Wald test *P* = 0.033) in the expected direction, such that chicks from early eggs are 0.003 ± 0.001 g heavier per egg rank at day 0 (*P* = 0.011). This is nonsignificant by the end of ontogeny (–0.000 ± 0.008 g per rank, *P* = 0.996).

### CHICK SURVIVAL

The fixed effects included in the model of survival are summarized in Table 3. There were no significant effect of any form of coadaptation on survival (Wald test: PO coadaptation *P* = 0.143; SPO coadaptation *P* = 0.702; sibling coadaptation *P* = 0.183). In each case, nonsignificant family maladaptation was seen at day 0 and nonsignificant family coadaptation seen at day 15.

**Table 3:** Fixed effect results for the effect of treatment on the survival of chicks over ontogeny. The mechanisms by which crossed offspring may lose fitness have been fitted as parent‐offspring coadaptation (PO), sibling coadaptation (S), and sibling‐mediated parent–offspring coadaptation (SPO). In addition, odd nests were fitted as a separate factor, as were uncrossed individuals in the full treatment

	Mean	l−95%	u–95%	*P*(MCMC)	*P* (Wald)
Day 0	14.214	8.775	19.397	<0.001	
Day 1	14.472	9.513	19.619	<0.001	
Day 3	13.892	9.423	18.372	<0.001	
Day 6	16.541	12.744	20.670	<0.001	
Day 9	17.023	13.177	20.619	<0.001	
Day 12	18.644	14.732	22.600	<0.001	
Day 15	20.633	16.335	25.306	<0.001	
Time	−0.067	−3.067	3.505	0.960	
Time: Day	0.048	−0.241	0.321	0.737	0.738
Sex (M)	−0.013	−0.251	0.218	0.948	
Sex (M): Day	0.002	−0.019	0.024	0.880	0.883
Year 2011	0.258	−1.019	1.675	0.707	
Year 2011: Day	−0.333	−0.452	−0.227	<0.001	<0.001
Year 2012	−0.364	−1.544	1.080	0.561	
Year 2012: Day	−0.107	−0.214	−0.011	0.043	0.039
Year 2013	0.872	−0.692	2.497	0.294	
Year 2013: Day	−0.072	−0.198	0.046	0.250	0.245
Year 2014	0.734	−0.744	2.339	0.353	
Year 2014: Day	−0.111	−0.239	0.009	0.076	0.084
Year 2015	−0.072	−1.407	1.305	0.905	
Year 2015: Day	−0.190	−0.300	−0.080	<0.001	<0.001
Clutch Size	−0.080	−0.313	0.144	0.510	
Clutch Size: Day	−0.009	−0.028	0.010	0.314	0.332
Hatch Date	−0.075	−0.135	−0.009	0.017	
Hatch Date: Day	−0.013	−0.019	−0.008	<0.001	<0.001
Hatch Day (0‐1)	−1.277	−1.658	−0.939	<0.001	
Hatch Day (0‐1): Day	0.042	0.010	0.075	0.014	0.013
Hatch Day (1‐3)	−3.402	−4.153	−2.664	<0.001	
Hatch Day (1‐3): Day	0.141	0.075	0.218	<0.001	<0.001
Egg Rank	−0.047	−0.121	0.029	0.235	
Egg Rank: Day	0.007	−0.000	0.015	0.051	0.059
Odd nest	−0.013	−1.709	1.632	0.984	
Odd nest: Day	−0.065	−0.191	0.065	0.350	0.338
PO	−0.213	−0.507	0.094	0.187	
PO: Day	0.021	−0.008	0.047	0.151	0.143
S	−0.789	−1.869	0.303	0.159	
S: Day	0.068	−0.032	0.169	0.190	0.183
SPO	−0.364	−2.136	1.588	0.703	
SPO: Day	0.029	−0.117	0.180	0.685	0.702

In the case of the PO coadaptation, chicks that remained with their biological parents had a nonsignificant decrease in survival at day 0 (–0.213 probits [–0.507 to 0.094] *P* = 0.187), and a nonsignificant increase at day 15 (0.095 probits [–0.087 to 0.281] *P* = 0.312). The sibling coadaptation effect at day 0 (–0.789 probits [–1.869 to 0.303] *P* = 0.159) and day 15 (0.231 probits [–0.461 to 0.902] *P* = 0.506) were similar, as were the SPO coadaptation effects; day 0 (–0.364 probits [–2.136 to 1.588] *P* = 0.703) and day 15 (0.070 probits [–1.544 to 1.554] *P* = 0.933). Chicks in odd nests did not differ from other chicks in their survival probability (Wald test *P* = 0.338; –0.013 probits [–1.709 to 1.632] *P* = 0.984 at day 0; –0.995 probits [–2.494 to 0.500] *P* = 0.193 at day 15). As with mass, no bias‐correction terms were large or significant and were dropped for clarity.

Time, sex, and clutch size had no significant effects on survival, but there were significant effects of year, hatch date, and hatch day within the clutch (Table 3). Egg rank had a nonsignificant effect on survival (Wald test *P* = 0.059) with chicks from higher rank eggs (early laid) having nonsignificantly reduced survival probabilities at day 0 (–0.047 probits [–0.121 to 0.029] *P* = 0.235) and increased survival probabilities by day 15 (0.061 probits [0.005 to 0.118] *P* = 0.039).

### ADULT SURVIVAL AND FECUNDITY

The number of adults that died or survived after either 2014 or 2015 for each treatment is shown in Table 4, and the results from the model of adult survival are in Table 5. For females, there is a nonsignificant positive effect of the full treatment on survival (0.110 probits [–0.410 to 0.625] *P* = 0.673), and a nonsignificant negative effect of the partial treatment (–0.216 probits [–0.763 to 0.289] *P* = 0.417) compared to controls. Females raising nests classed as odd have nonsignificantly lower survival (–0.339 probits [–0.969 to 0.229] *P* = 0.271). For males, there is also a nonsignificant positive effect of the full cross‐fostering treatment (0.227 probits [–0.374 to 0.822] *P* = 0.472), and a nonsignificant negative effect of the partial cross‐fostering treatment (–0.024 probits [–0.621 to 0.534] *P* = 0.946). There was also a nonsignificant positive effect of raising odd broods (0.093 probits [–0.565 to 0.835] *P* = 0.788). Survival was higher in 2015 than 2014 (0.303 probits [0.025 to 0.583] *P* = 0.035). It should be noted, however, that the credible intervals are quite wide, and the upper 95% intervals are compatible with strong effects of coadaptation on parental fitness.

**Table 4:** The number of adults that died or survived when exposed to each of the treatments in 2014

	2014		2015	
Treatment	Died	Survived	Died	Survived
Control	26	15	18	19
Full	18	22	24	21
Partial	31	13	27	24
Odd	21	7	9	11

**Table 5:** The effects of treatment on survival of adults to the subsequent breeding season

	mean	l–95%	u–95%	*P*(MCMC)
Sex F	−0.233	−0.624	0.199	0.261
Sex M	−0.418	−0.889	0.024	0.084
Year 2015	0.303	0.025	0.583	**0.035**
Male: Odd	0.093	−0.565	0.835	0.788
Male: Full	0.227	−0.374	0.822	0.472
Male: Partial	−0.024	−0.621	0.534	0.946
Female: Odd	−0.339	−0.969	0.229	0.271
Female: Full	0.110	−0.410	0.625	0.673
Female: Partial	−0.216	−0.763	0.289	0.417

**Figure 4:**
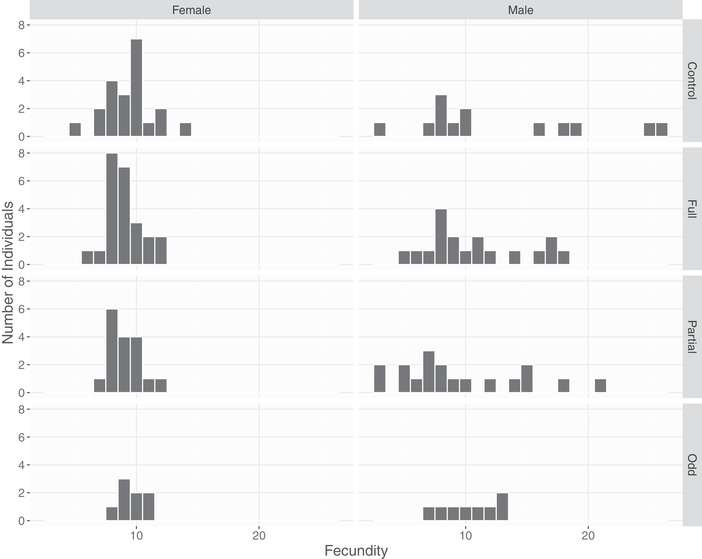
The fecundities of adults in the year following the experiment, split by sex and the treatment they received.

The fecundities of adults that survived from 2014 or 2015 in the different treatments are shown in Figure 4, and the results of the model are shown in Table 6. For females there were nonsignificant negative effects of raising a fully crossed brood (–0.018 log eggs [–0.120 to 0.074] *P* = 0.710) or a partially crossed brood (–0.051 log eggs [–0.150 to 0.056] *P* = 0.344) compared to controls. There was also a nonsignificant increase in fecundity in those that had odd nests (0.041 eggs [–0.103 to 0.169] *P* = 0.537). For males, there was a nonsignificant decrease in fecundity for the full treatment (–0.076 log eggs [–0.412 to 0.256] *P* = 0.659), or the partial treatment (–0.268 log eggs [–0.647 to 0.066] *P* = 0.122). The effect of raising odd nests was also negative and nonsignificant (–0.097 log eggs [–0.534 to 0.327] *P* = 0.662). Fecundity was significantly higher in 2016 than 2015, resulting in greater future fecundity of the 2015 cohort (0.127 log eggs [0.051 to 0.200] *P* < 0.001).

**Table 6:** The effects of treatment on fecundity of the surviving adults in the subsequent breeding season

	mean	l–95%	u‐95%	*P*(MCMC)
Sex F	2.155	2.080	2.234	<**0.001**
Sex M	2.313	2.046	2.581	<**0.001**
Year 2015	0.127	0.051	0.200	<**0.001**
Male: Odd	−0.097	−0.534	0.327	0.662
Male: Full	−0.076	−0.412	0.256	0.659
Male: Partial	−0.268	−0.647	0.066	0.122
Female: Odd	0.041	−0.103	0.169	0.537
Female: Full	−0.018	−0.120	0.074	0.71
Female: Partial	−0.051	−0.150	0.056	0.344

## Discussion

By carrying out a randomized cross‐fostering experiment, this study aimed first to test for evidence of PO coadaptation in blue tits, and secondly whether sibling coadaptation and/or SPO coadaptation exist. PO coadaptation is primarily detected by comparing the fitnesses of crossed and uncrossed offspring, irrespective of the type of nest (full or partial) in which they were raised in (comparisons 1 and 2 in Figure 1), with the expectation that crossed offspring will have reduced fitness. We did not find a reduction in offspring mass or survival when offspring were raised by foster rather than biological parents, and instead saw a slight increase in mass when offspring were cross‐fostered. This is in contrast to the results found by Hinde et al. (2010), who found evidence of PO coadaptation through growth costs to offspring.

The power to detect PO coadaptation effects on offspring was high (changes in mass of <1% could have been detected) and so we are fairly confident that if PO coadaptation does exist, the effect is likely to be small. Although we have framed our results in the context of genetic coadaptation, Hinde et al. (2010) considered coadaptation from the idea that signals within eggs (prenatal maternal effects) may generate correlations between parents and offspring (see Giordano et al. 2014, also). Components placed within eggs may act as signals or cues of parental ability, leading to modulation of offspring behavior in response to the prenatal parental environment (e.g., Hinde et al. 2009; Paquet et al. 2015, although see Estramil et al. 2017). Although this involves a fundamentally different mechanism for matching offspring and parental phenotype, the predicted fitness outcomes are the same as those under genetic coadaptation, and so we believe that our study also rejects a major role for between‐brood anticipatory parental effects in blue tits. Likewise, antogonistic behavior of parents (Murie et al. 1998) or siblings (Boncoraglio and Saino 2008) to non‐kin give the same predicted fitness outcomes as family coadaptation, and so this work casts doubt on the ability of blue tits to recognize kin based on genetic or prenatal cues. This conclusion is largely in‐line with the findings of relevant studies summarized in the introduction.

Our empirical finding that there is little genetic coadaptation is also mostly in‐line with what would be expected theoretically. In order for PO coadaptation effects on offspring to exist, parental‐effect alleles and the alleles determining the affected offspring traits must be segregating and the two sets of alleles must either be the same (i.e., pleiotropic) or be in linkage disequilibrium. There is direct evidence that parental effect alleles segregate in wild systems (Wilson et al. 2005). There is also indirect evidence whereby traits thought to be responsible for parental effects (e.g., provisioning rates) have been shown to be heritable, in long‐tailed tits (MacColl and Hatchwell 2003), house sparrows (Dor and Lotem 2010), and burying beetles (Walling et al. 2008). Consequently, the available evidence suggests that a lack of segregating alleles is unlikely to limit the potential for PO coadaptation. However, the conditions under which parental effect and offspring alleles would be non‐independent in order to generate the appropriate genetic correlations seem harder to fulfil. Although linkage disequilibrium may be strong enough to allow PO coadaptation if generated by population structure (Zakas et al. 2018) or speciation (Capodeanu‐Nägler et al. 2018), within‐population PO coadaptation would be harder to achieve. Indeed, Santure et al. (2013) have shown that there is low linkage disequilibrium in the great tit, and assuming similar patterns hold in blue tits, the potential for such coadapted gene complexes to evolve in this species may be limited, and the lack of evidence for PO coadaptation unsurprising (Lande 1980; Hadfield 2012). Pleiotropic effects are a more likely route by which genetic correlations, and thus PO coadaptation, could occur but the developmental pathways of the parental and offspring traits that mediate any coadaptation (such as provisioning and begging) are likely to be quite different, limiting the opportunity for pleiotropy. Indeed, genetic correlations between begging and provisioning were not detected in blue tits (Lucass et al. 2015a), although the power of this study was low. The evolution of maternal effects that match offspring and parental phenotype may then provide a more plausible route by which co‐adaptation could arise (Hinde et al. 2010; Giordano et al. 2014). However, evidence that mothers prime their offspring for the environmental conditions that they themselves experienced seems to be generally weak (Uller et al. 2013), although forms of coupling other than parent–offspring environment matching may be more important but remain less well tested (Burgess and Marshall 2014).

If siblings affect each other through non‐excludable (in the economic sense, in which non‐paying consumers cannot be prevented from accessing a resource) parental manipulation, then using the test for PO coadaptation alone would miss this mechanism, which we have called sibling‐mediated parent–offspring coadaptation. Under this scenario, we would expect to find partially cross‐fostered nests to be intermediate between control and fully cross‐fostered nests, as some biological offspring of the parents remain at that nest. Although the power to detect this type of effect was lower than for PO coadaptation, we found little evidence for SPO coadaptation. In addition, if direct interactions between siblings are present, sibling coadaptation may exist, and we would expect chicks in partial cross‐fosters to do worse than control and fully cross‐fostered chicks. Again, we found little evidence for sibling coadaptation, thus individuals do not seem to benefit from being with siblings, either through direct or indirect mechanisms. These mechanisms require that sibling genetic effects exist, for which there is only rudimentary evidence (Ashbrook et al. 2017), although as with offspring genetic effects (Agrawal et al. 2001) unequivocal evidence is hard to obtain and few have tried. Even if heritable traits that had effects on siblings were identified, it is hard to imagine what sort of traits could plausibly generate sibling coadaptation. However, sibling‐mediated parent–offspring coadaptation seems more plausible in systems in which parents respond to characteristics of the group rather than the individual, and may prove to be a fruitful avenue for future research.

As well as considering potential effects of coadaptation on offspring, we tested whether there were effects of the cross‐fostering treatments on parental fitness. Under PO and SPO coadaptation, we predict that adults raising foster chicks should suffer compared to controls, and that parents raising fully crossed nests should suffer a greater fitness cost than those raising partially crossed nests, due to the presence of some biological offspring in partially treated nests. However, if those raising partially crossed broods suffer more than those raising fully crossed broods, this may imply that parents bear the effects of sibling conflict (even if those offspring themselves do not appear to show costs of this). However, there appears to be no cost to either females or males when raising fully cross‐fostered or partially crossed broods and so no evidence of parent–offspring or sibling‐mediated parent–offspring coadaptation on parental fitness. This might be expected from the traditional veiwpoint of parents as actors, rather than recipients, in which there is little scope for offspring behaviors to impact on parental fitness. Indeed attempts to manipulate parental care in birds (such as brood‐size manipulations) find at best modest effects on parental fitness, which are often explained by parents passing the costs of parental care onto their passive offspring (Santos and Nakagawa 2012).

Winney et al. (2015) showed an apparent increase in the fitness of house sparrow chicks when raised with and by non‐relatives, but attributed this to nonrandom assignment of chicks and nests to cross‐fostering treatment. Although we used a balanced randomized design in order to reduce this type of problem, crossing eggs on the day they were laid means that crossed eggs tended to be earlier in the laying sequence, when all females were still laying and eggs could be switched. Early eggs tend to hatch earlier (Hadfield et al. 2013b) and are more likely to have been sired by extra pair males (Magrath et al. 2009), both of which are likely to favor higher body masses and survival. We tried to control for this in our analyses by using hatching time and egg rank as predictors. However, as multiple chicks hatch between visits to the nests, we are often not able to assign a chick to the exact egg from which it hatched. In lieu of this information, we used mean egg ranks, which may not be able to completely compensate for this bias. Nevertheless, the mean egg ranks of crossed and uncrossed eggs and chicks are similar and the effect of egg rank in the model, although significant, is small once hatching time is accounted for. Consequently, although we expect that this (unavoidable) inadequacy in our design is likely to bias the estimates of coadaptation, we believe these biases are likely to be small.

A common alternative to our design is to cross chicks, or eggs after clutch completion, which would simplify both the logistics and the statistical analyses. However, in partial cross‐fostering, this generates age‐related size differences between the two half‐broods due to differences in hatching time (Hadfield et al. 2013a), particularly in egg swaps post‐clutch completion because of differential incubation prior to cross‐fostering (Hadfield et al. 2013b). Because crossed chicks are as equally likely to hatch earlier as later, this would still provide a valid comparison of crossed and non‐crossed chicks *within* partial crossed nests, and therefore, provide a valid estimate of PO coadaptation. However, such a protocol would compromise any “between” nest comparisons of chicks in partial crossed nests and those in control nests. Because partial nests will have greater hatching asynchrony and the chicks will be on average younger (typically measurements are taken a set number of days after the first chick hatches), chicks from partially crossed nests will tend to be smaller and have higher mortality than those from control nests. This would occur irrespective of whether they are crossed or not, and would be incorrectly attributed to S or SPO coadaptation, or kin recognition of siblings (Boncoraglio and Saino 2008).

The cross‐fostering design in this study is the first to compare both fully and partially cross‐fostered broods, along with appropriate control broods in a wild population (see Heim et al. 2012 for a study in domestic pigs, with similar findings). We used these comparisons to test for parent–offspring coadaptation, and sibling effects. We did not find any evidence for parent–offspring coadaptation, either through direct interactions or the effect of siblings, nor did we find any evidence for sibling coadaptation. Because the expected outcomes under kin‐recognition, or environmental matching of offspring and parental phenotype, are identical the evidence for these processes is also weak. The question remains as to whether this result is general, and the mixed results from previous studies reflect Type I errors, or whether real heterogeneity exists between species. The large number of reciprocal cross‐fostering studies by quantitative geneticists, few of which estimate the differences between crossed and non‐crossed chicks, would be a valuable resource for obtaining a broader evidence‐base for PO coadaptation. With archived data it may be possible to revisit these studies, and future studies should be encouraged to estimate and report this important parameter.

Associate Editor: J. McGlothlin

Handling Editor: P. Tiffin
